# Temporary microglia-depletion after cosmic radiation modifies phagocytic activity and prevents cognitive deficits

**DOI:** 10.1038/s41598-018-26039-7

**Published:** 2018-05-18

**Authors:** Karen Krukowski, Xi Feng, Maria Serena Paladini, Austin Chou, Kristen Sacramento, Katherine Grue, Lara-Kirstie Riparip, Tamako Jones, Mary Campbell-Beachler, Gregory Nelson, Susanna Rosi

**Affiliations:** 1 Department of Physical Therapy and Rehabilitation Science, University of California, San Francisco, CA USA; 2 Brain and Spinal Injury Center, University of California, San Francisco, CA USA; 3 Department of Basic Sciences, Division of Biomedical Engineering Sciences, Loma Linda University, Loma Linda, CA USA; 4 Department of Neurological Surgery, University of California, San Francisco, CA USA; 5 Weill Institute for Neuroscience, University of California San Francisco, San Francisco, CA USA; 6 Kavli Institute of Fundamental Neuroscience, University of California San Francisco, San Francisco, CA USA

## Abstract

Microglia are the main immune component in the brain that can regulate neuronal health and synapse function. Exposure to cosmic radiation can cause long-term cognitive impairments in rodent models thereby presenting potential obstacles for astronauts engaged in deep space travel. The mechanism/s for how cosmic radiation induces cognitive deficits are currently unknown. We find that temporary microglia depletion, one week after cosmic radiation, prevents the development of long-term memory deficits. Gene array profiling reveals that acute microglia depletion alters the late neuroinflammatory response to cosmic radiation. The repopulated microglia present a modified functional phenotype with reduced expression of scavenger receptors, lysosome membrane protein and complement receptor, all shown to be involved in microglia-synapses interaction. The lower phagocytic activity observed in the repopulated microglia is paralleled by improved synaptic protein expression. Our data provide mechanistic evidence for the role of microglia in the development of cognitive deficits after cosmic radiation exposure.

## Introduction

The next era of human exploration will be as challenging as it is exciting. As we seek to explore planets beyond our own, both the distances traversed and the difficulties faced during the voyage will be new to humankind. Long space missions, such as the upcoming Mars journey, require a clear understanding of the effects of space exposure on the mental and physical functional capabilities of astronauts. The psychological stressors that astronauts face during space travel are powerful and varied, including social isolation, altered sleep patterns, and physical space limitations^1^. This is coupled with the direct impact of the adverse environment of space, which includes exposure to galactic cosmic rays (GCR). GCR are composed of protons, helium nuclei, and high charge and energy nuclei, which can alter cellular machinery and function. The short and/or long-term effects of GCR exposure are largely unknown. It is estimated that during deep space missions astronauts will experience 10-fold higher GCR exposure than when on the international space station. Two major contributors of this exposure will be from protons and helium nuclei, therefore understanding of the effects of these particles is vital for mission success^1–5^.

Only in the last decade research has started to focus on how GCR can impact the central nervous system (CNS), with much effort devoted to developing mitigating factors that could prevent or rescue the loss of cognitive functions. Initial studies from our group and others have developed rodent models to study the effects of individual particle exposure. Our group found that exposure to protons, or protons coupled with iron, can cause acute as well as persistent cognitive loss as measured by the novel object recognition task (NOR)^4^. Radiation-induced cognitive loss is mediated in part by changes in/by the hippocampus. Importantly we have found that GCR exposure can alter the expression of the plasticity-related immediate early gene *Arc* and hippocampal networks mediating spatial memory^4,6–8^. Other recent rodent studies have reported behavioral impairments coupled with synapse alterations following exposure to different GCR particles- specifically titanium or oxygen^9–11^. Notably, the behavioral and synaptic alterations observed previously correspond with a modified inflammatory response^4^ and enhanced microglia activation up to 12 months after exposure^9,12^. Taken together, these studies suggest that GCR exposure can impact both neuronal and microglial cell function when radiation-induced cognitive deficits are measured.

Microglia are the resident macrophages and the main immune component in the brain, accounting for 10–15% of all brain cells. They constantly survey for signals of injury/infection; quickly moving toward affected sites upon activation, acting as key mediators of neuroinflammatory processes. It has long been established that microglia can passively regulate neuronal health through the release of cytokines and chemokines, however, more recent reports found direct effects of microglia on synapse function^13,14^. Microglia mediated synaptic interaction can be dependent on the complement cascade, which includes C1q and C3. The complement cascade, originally thought only to regulate pathogenic immune responses and phagocytosis, has been recently found to mediate synaptic elimination during brain development and in neurodegenerative diseases. It has been reported that inhibition of C1q or C3 reduces synapse loss, and pharmacological depletion of microglia prevents neuronal and synapse losses in Alzheimer’s models^15,16^. In line with these results, we and others have shown that microglia depletion at the time of radiation can prevent cognitive decline and dendritic spine loss in the hippocampus after brain only irradiation^17,18^.

It is estimated that during deep space missions, each cell in the body will be traversed by a helium ion approximately once every three weeks^3–5,19,20^ yet, despite this risk, there remains a paucity of studies investigating its effects on CNS function. In this study, we hypothesized that helium exposure affects memory function and that microglia activation plays a critical role in the development of cognitive deficits after helium exposure. To test these notions, we first examine the early and late effects of helium exposure on cognitive function. Second, we test whether temporary microglia depletion after helium exposure will prevent the development of cognitive deficits. Finally, we determine the functional characteristics of the repopulating microglia to understand their direct role on cognition.

## Results

### Radiation exposure paradigm and experimental design

**Figure 1:**
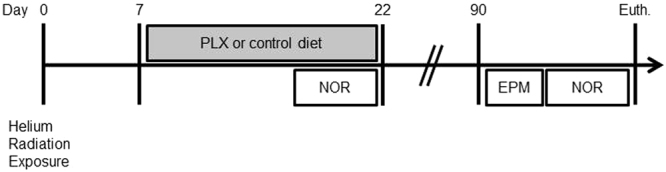
Experimental Design. Animals were exposed to helium radiation (0, 15, 50, 100 cGy) on day 0. Diet changes (+/−PLX) and behavioral analysis are shown relative to radiation exposure. NOR = Novel Object Recognition. EPM = Elevated Plus Maze.

Twenty-one week old male C57Bl6J mice were exposed to different doses of helium (0, 15, 50, 100 cGy) at Brookhaven National Laboratory. Seven days after radiation exposure mice were switched to PLX5622-1200ppm (PLX) or control diet for 15 days, (Fig. 1**)**. The PLX diet is composed of a CSF1-R inhibitor that induces depletion of microglia within 3 days (~90%), and sustains the depleted state throughout the duration of treatment^17,21^. Upon returning to normal diet microglia repopulation occurs within seven days^21–23^. Behavioral readouts for recognition memory and anxiety were measured early after radiation, during microglia depletion (18–21 days), and long-term post radiation (90–100 days, Fig. 1**)**.

### Brief microglia depletion fully rescued the development of long term radiation-induced memory deficits

**Figure 2:**
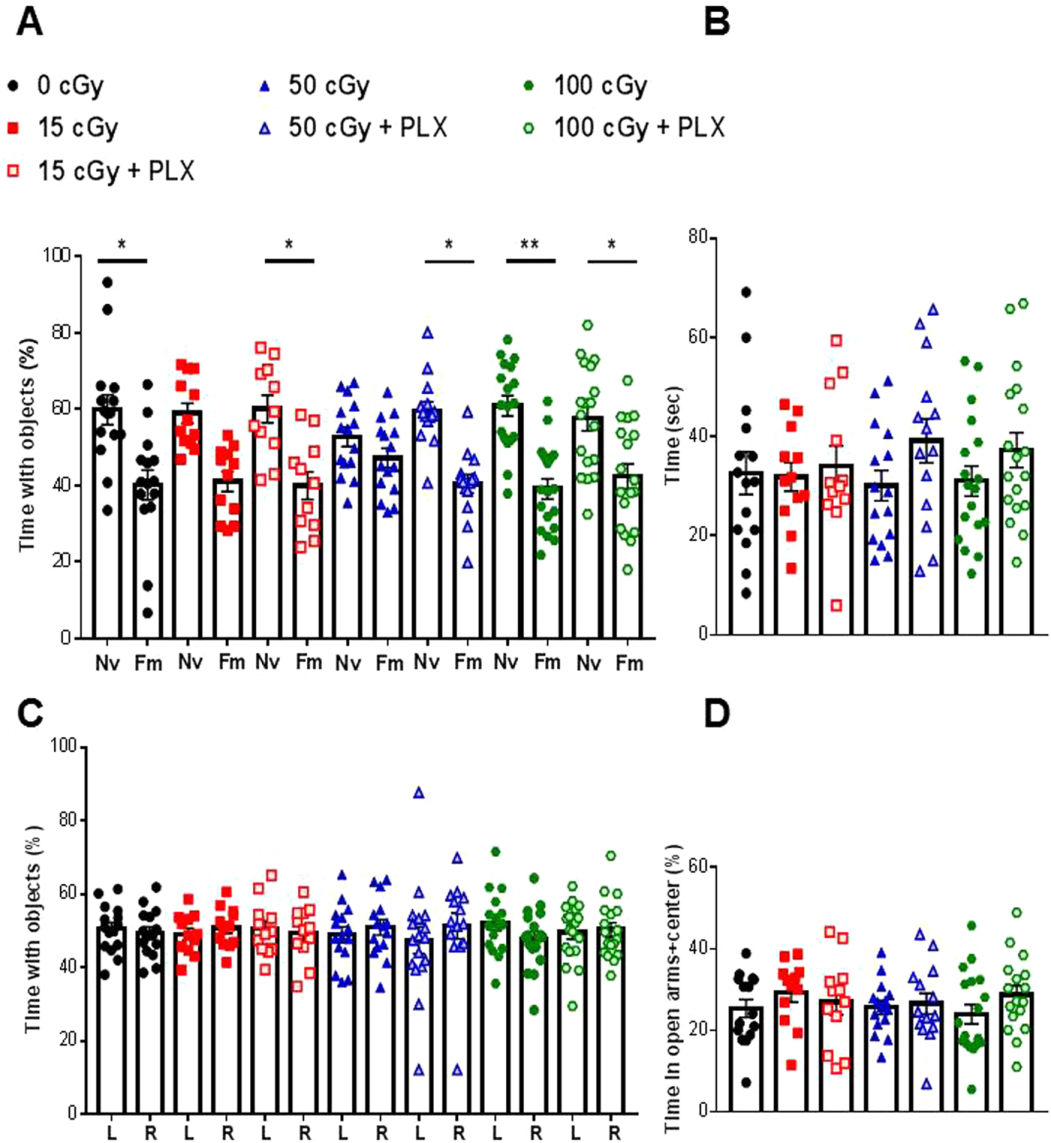
Radiation-induced memory impairments rescued by PLX treatment. Animals were exposed to helium radiation (0, 15, 50, 100 cGy). Beginning 90 days later, animals were tested for memory deficits or anxiety-like behavior. (**A**) Memory deficits were measured by novel object recognition. Animals were exposed to two identical object, 24 hrs later the animals are exposed to one familiar object and one novel object. Memory deficits are calculated by a deficit in distinguishing the new object. Nv = novel. Fm = Familiar. Two-way repeated measured ANOVA found a significant group (p < 0.0001) and discrimination effect (p < 0.0001). Sidak post hoc analysis revealed differences in discrimination effects. Animals exposed to 15 or 50 cGy of helium radiation were unable to distinguish the objects, denoting memory impairment. (**B**) Total exploration time with both objects is depicted per group. (**C**) Arena side preference is determined during the familiarization phase. In which animals are exposed to two identical objects. Time spent exploring each object is measured. L = object on the left side of the arena, R = object on the right side of the arena. (**D**) Anxiety-like behavior was measured by time spent in the open arms in the elevated plus maze. A one-way ANOVA did not reveal any significant differences between groups. *p < 0.05, **p < 0.01. Individual animal scores represented in dots, bars depict group mean and SEM. N = 12–18 each group.

To study the possible effects of helium irradiation on memory we used the novel object recognition task (NOR). In this task the animal’s preference for the novel object serves as the gold standard measure of recognition memory. We investigated the effect of helium exposure on early (18 + days post irradiation, during PLX or control diet) and late (90 + days post radiation) memory response. No memory impairments were detected early after exposure in any of the groups, (Supplemental Fig. 1). At late time points (90 + days post helium exposure, Fig. 1) mice exposed to either 15 or 50 cGy of helium were unable to distinguish the novel and familiar objects denoting deficits in recognition memory, (Fig. 2A, red squares and blue triangles). Strikingly, when either group was treated with PLX after irradiation, the long-term memory impairments were no longer observed, (Fig. 2A, red shaded squares or blue shaded triangles). No memory impairments were measured in any other group, (Fig. 2A). Importantly, no differences were measured in total exploration time (Fig. 2B) or object preference during identical object exploration (Fig. 2C).

Furthermore, we analyzed anxiety-like behavior by the elevated plus maze (EPM) at 90 days post radiation exposure. No groups displayed any anxiety-like behavioral phenotypes (Fig. 2D). Thus, poor performance in the NOR task was not a result of inactivity or anxiety-like behavior. Taken together these data suggest that temporary microglia depletion acutely after radiation exposure could be a potential therapeutic option for treating long-term radiation-induced memory impairments.

### Radiation-induced long-term changes in inflammatory markers

**Figure 3:**
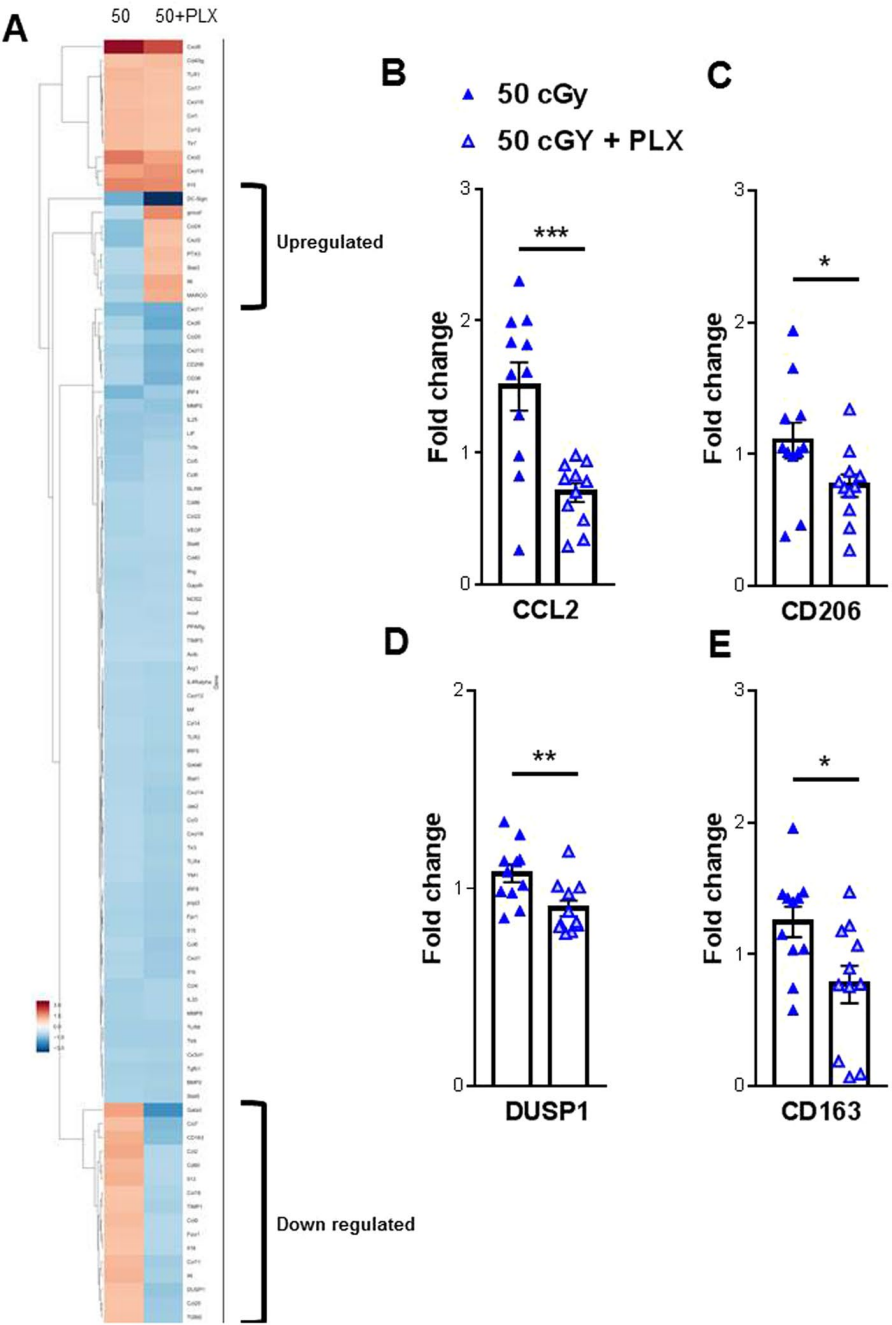
Radiation-induced gene changes. (**A**) Gene-expression changes were measured by a 96 chemokine and cytokine target gene array. qPCR analysis found four genes with significant differences between the 50 cGy and 50 cGy + PLX group: (**B**) CCL2 (**C**) CD206 (**D**) DUSP1 and (**E**) CD163. Unpaired Student t-test revealed significant differences between groups. *p < 0.05, **p < 0.01, ***p,0.001. Individual animal scores represented in dots, bars depict group mean and SEM. N = 11–12 each group.

Gene array profiling was performed to examine the effect of radiation exposure and/or transient microglia depletion upon the neuroinflammatory response. Using hippocampal lysates from 0 cGy, 50 cGy and 50 cGy + PLX treated mice we performed a 96 chemokine and cytokine gene array assay to investigate possible genetic signatures responsible for radiation-induced behavioral effects, (Fig. 3A). While the majority of the gene profiles were similar between the 50 cGy and 50 cGy + PLX groups, we did observe a subset of genes in which PLX treatment down-regulated expression and a second subset in which PLX treatment upregulated gene expression (Fig. 3A). Further validation by qPCR on targets from these subsets revealed four targets with significant group effects. CCL2, CD206, CD163 and DUSP1 were all down-regulated in the 50 cGy + PLX group when compared to 50 cGy alone (Fig. 3B-E). We also investigated if inflammatory signatures differed in two groups that did not display behavior deficits (100 cGy and 100 cGy + PLX). We found no changes in CCL2 and DUPS1 when comparing 100 cGy to 100 cGy + PLX. Interestingly, CD163 was still down-regulated following PLX treatment, whereas CD206 was increased with PLX treatment. These data demonstrate that brief microglia depletion after helium irradiation alters long-term neuroinflammatory processes. Furthermore, these data suggest that the repopulated microglia and associated microenvironment exhibit altered genetic and functional phenotype.

### Microglia and peripheral macrophage numbers were not affected by whole body helium irradiation followed by transient microglia depletion

**Figure 4:**
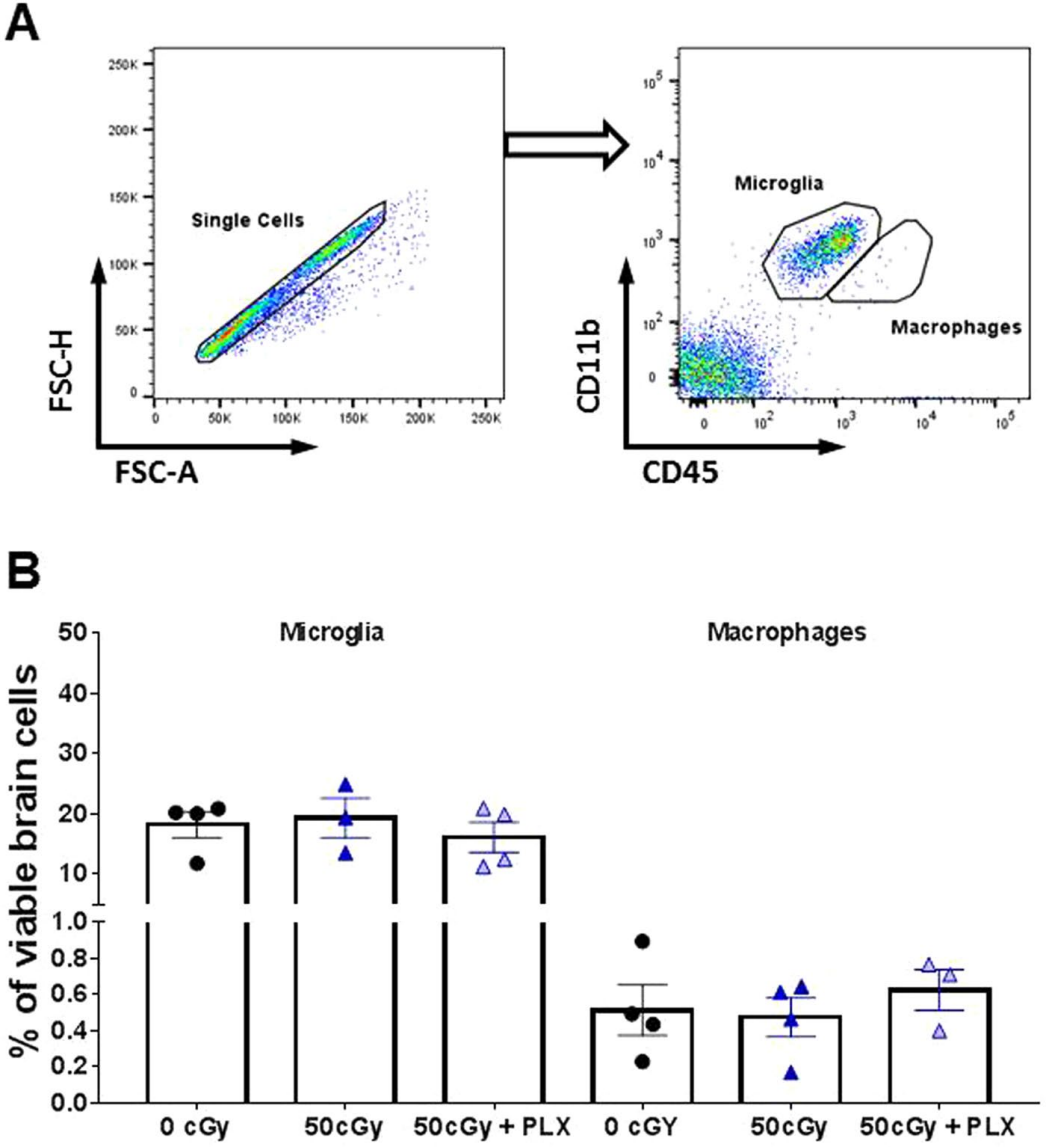
Flow cytometry analyses of microglia and periphery originated cells in the CNS. (**A**) Gating for microglia and peripherally derived macrophage population analysis. Microglia (CD11b^+^CD45^low^) and peripherally derived macrophage (CD11b^+^CD45^high^) populations were identified by their differentially expressed cell surface markers. (**B**) A graph to show the relative populations of microglia and peripheral cells in the CNS. Data are presented as dot plots (mean +/−SEM) to show percentage of either population in all viable cells. A one-way ANOVA did not reveal any significant differences between groups. N = 4 each group.

To determine the impact of temporary microglia depletion and/or radiation exposure on repopulation late after exposure (90 + days) in the brain we used flow cytometry. We did not observe any differences across any treatment groups in either microglia (CD11b^+^CD45^low^) or peripherally derived macrophage (CD11b^+^CD45^high^) numbers (Fig. 4). These results suggest that whole-body helium irradiation does not affect microglia or peripheral macrophage numbers. Furthermore, it demonstrates that after removal from the temporary PLX diet, microglia and macrophages repopulate to levels similar to untreated animals.

### Temporary microglia depletion decreases complement receptor C5aR in the repopulated microglia

**Figure 5:**
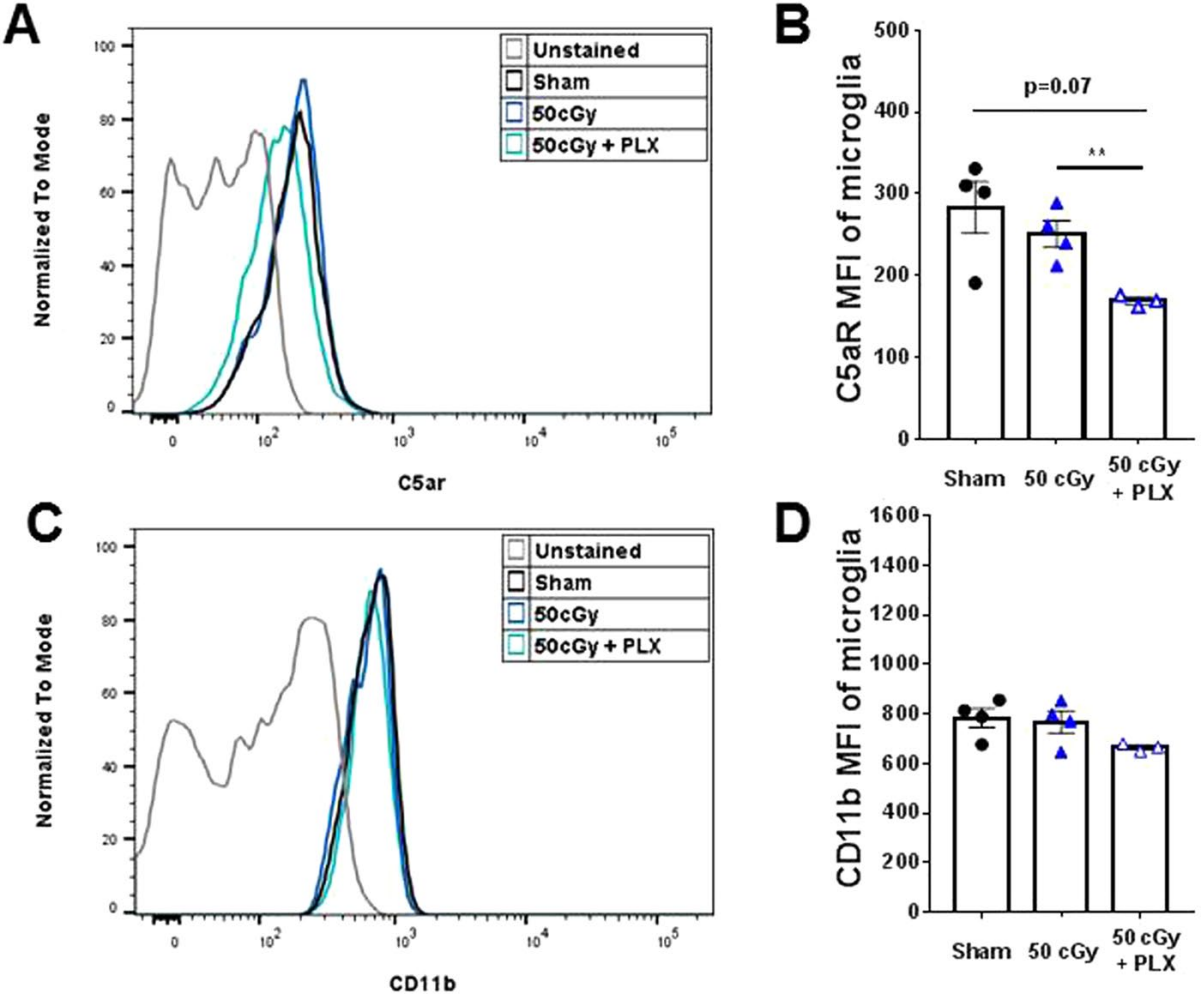
Analyses of complement markers in microglia. Mean fluorescence intensity (MFI) was used to compare the protein levels in microglia populations (CD11b^+^CD45^low^). (**A**,**B**) Comparison of C5aR MFI by surface staining on microglia. PLX5622 treatment after 50 cGy helium irradiation results in significantly reduced C5aR level compared to the 50 cGy-only group, **p < 0.01, One-way ANOVA with Sidak’s multiple comparisons test. (**C**,**D**) Comparison of CD11b MFI by surface staining on microglia. Statistics reveals no significant difference in CD11b (CR3A) levels across groups, but there is a trend of decreased MFI in the 50 cGy + PLX5622 group, p = 0.1096, unpaired student t-test. N = 3–4 each group.

Next, we investigated whether alterations in the repopulated microglia activity at late end points could be responsible for the rescue of radiation-induced memory deficits. One way microglia can impact neuronal function is through synaptic interaction. The complement system has been shown to be involved in microglia-mediated synaptic engulfment under both physiological and disease conditions^13–15,24–26^. We characterized microglia expression levels of two mediators of the complement pathway: C5aR and CD11b (CR3A) by flow cytometry. Radiation alone did not impact C5aR levels; however, the repopulated microglia in the 50 cGy + PLX group displayed significantly decreased expression levels of C5aR when compared to the 50 cGy group (Fig. 5A,B). CD11b levels were not significantly affected by helium irradiation or PLX treatment (Fig. 5C,D), but there was a trend of decreased expression in the 50 cGy + PLX group. These results demonstrate that the repopulated microglia have reduced complement receptor levels that correspond with improved recognition memory.

### Repopulated microglia following helium irradiation exposure have lower phagocytic activity

**Figure 6:**
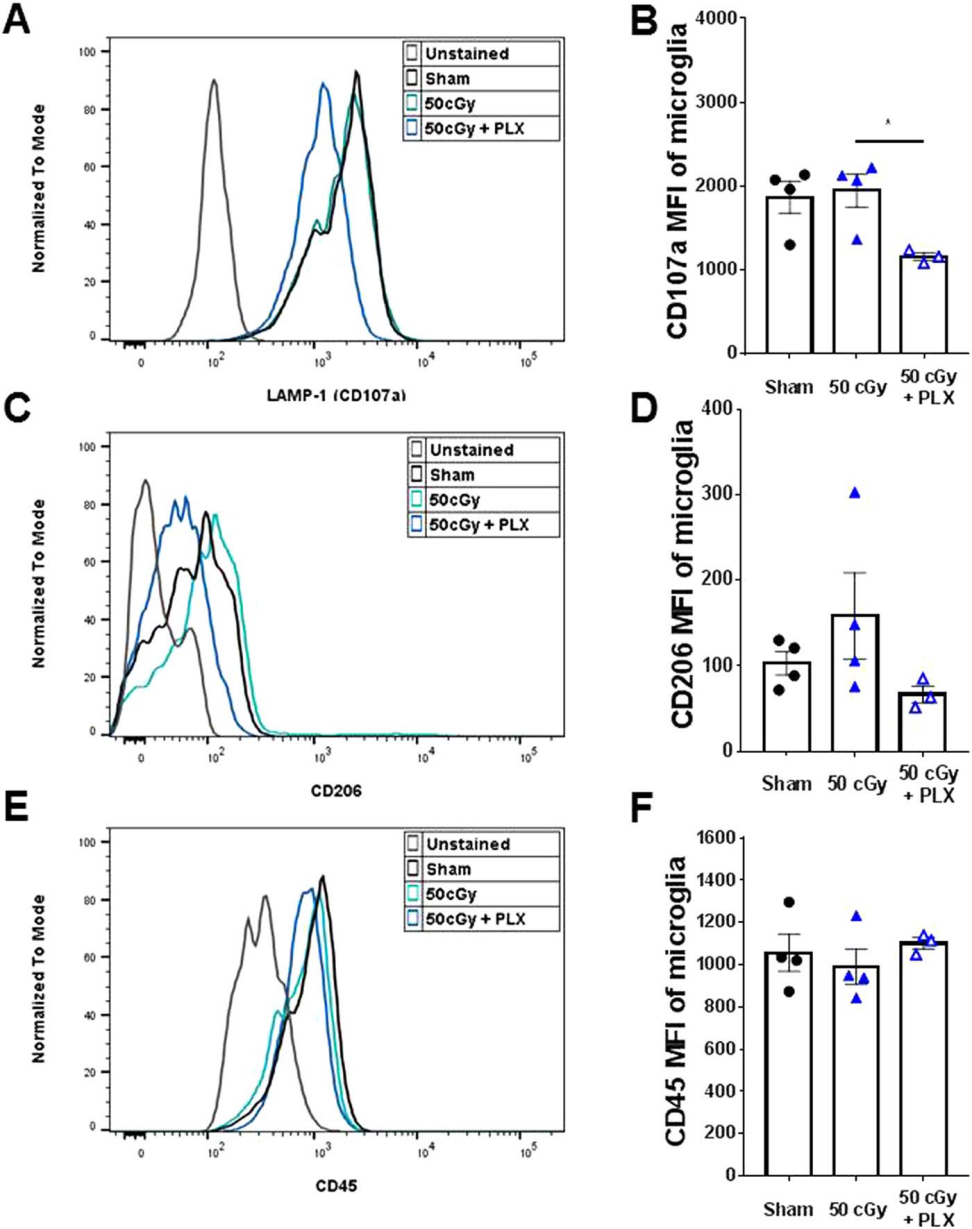
Analyses of phagocytic markers in microglia. MFI was used to compare the phagocytic marker levels in microglia. (**A**,**B**) Comparison of LAMP-1 (CD107a) MFI by intracellular staining in microglia. PLX5622 treatment caused significantly reduced CD107a compared to the 50 cGy group, *p < 0.05, One-way ANOVA with Sidak’s multiple comparisons test. (**C**,**D**) Comparison of CD206 MFI by intracellular staining in microglia. **(E**,**F)** Comparison of CD45 MFI by surface staining on microglia. There is no difference in microglial CD45 levels between groups. N = 3–4 each group.

To understand the long-term effects of helium irradiation and temporary microglia depletion on phagocytic activity we examined three key phagocytosis markers in microglia isolated from the brain: LAMP-1, CD206 and CD45. The repopulated microglia after 50 cGy + PLX treatment showed a significant decrease of LAMP-1 levels when compared to the 50 cGy group (Fig. 6A,B). No differences were measured in CD206 expression levels, however a trend downward in the 50 cGy + PLX group was measured (Fig. 6C,D). No differences in CD45 levels were measured between groups (Fig. 6E,F). These results suggest that the repopulated microglia have reduced phagocytic activity.

### Temporary microglia depletion alters synapse markers following helium irradiation exposure

**Figure 7:**
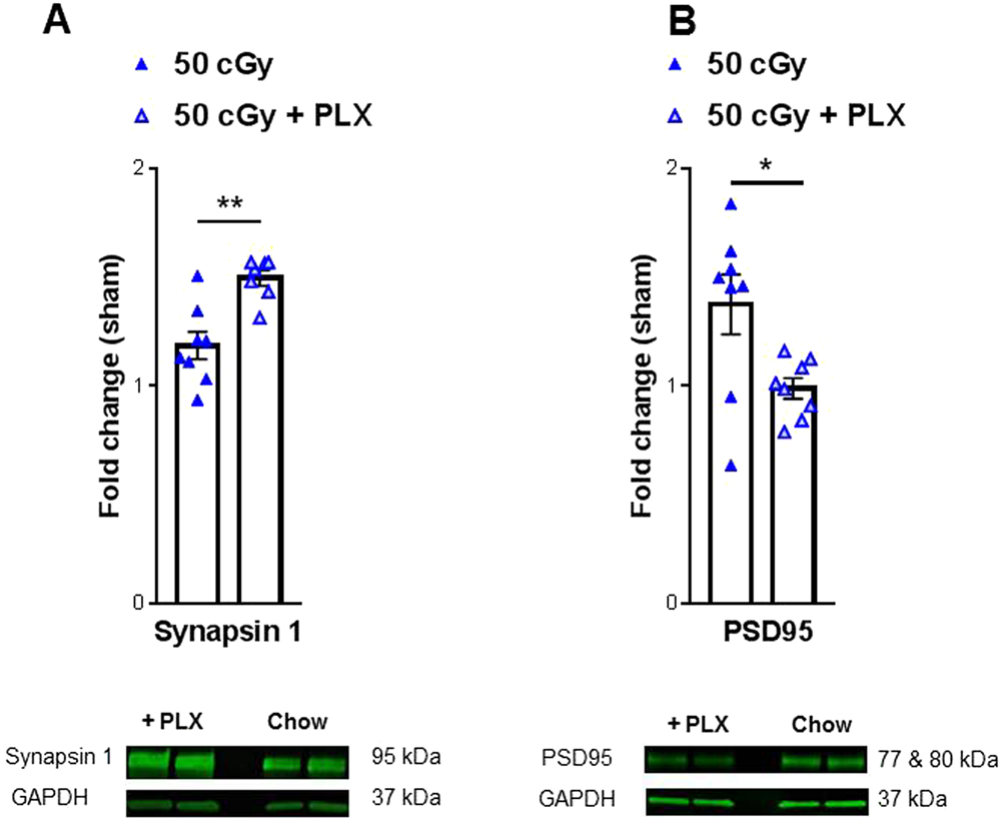
Investigation of neuronal stability markers. Western blot analysis of isolated prefrontal cortex tissues revealed changes in neuronal stability markers: synapsin 1 (**A**) and PSD95 (**B**). (**A**) A significant increase in synapsin 1 levels were observed in the 50 cGy + PLX group when compared to 50 cGy alone. Representative blot depicted below. (**B**) A significant decrease in PSD-95 levels were observed in the 50 cGy + PLX group when compared to 50 cGy alone. Representative blot depicted below. Full-length blots are presented in Supplemental Fig. 2. Samples were derived from the same experiment and were processed in parallel. Unpaired student t-tests reveal significant differences between groups. *p < 0.05, **p < 0.01. Individual animal scores represented in dots, bars depict group mean and SEM. N = 7–8 each group.

To investigate if alterations in the repopulated microglia phenotype relate to neuronal synapses levels we measured protein levels of two neuronal proteins: synapsin 1 and postsynaptic density protein 95 (PSD95). We observed significant increases in levels of the presynaptic protein, synapsin 1 in the 50 cGy + PLX when compared with the 50 cGy alone group, Fig. 7A and Supplemental Fig. 2. This is consistent with the hypothesis that less phagocytic repopulated microglia will have limited neuronal interactions. Conversely, we found a significant decrease in post synaptic marker, PSD95 levels in the 50 cGy + PLX when compared with the 50 cGy alone group, Fig. 7B and Supplemental Fig. 2. This is consistent with a previous report that measured increased PSD95 levels corresponding with decreased dendritic spine density^9^. Taken together these data suggest that the altered phenotype of the repopulated microglia could play a role in synaptic protein expression specifically in the proteins important for stability and synapse function.

## Discussion

Deep space journeys into the unknown are dangerous endeavors, the hope is that with preclinical studies we can both identify potential obstacles, as well as solutions for these problems. Here, we find long-term deficits in memory associated with whole body helium irradiation. For the first time we show that a brief microglia depletion after charged particle radiation exposure completely prevents memory deficits measured more than 90 + days later. Moreover, we found that the recovery in memory-deficit was at least in part due to: 1) a reduced neuroinflammatory milieu, 2) modifications in the repopulated microglia phenotype and 3) neuronal protein expression. Specifically, repopulated microglia displayed decreased expression of complement mediator C5aR and phagocytic marker LAMP-1. Cognitive rescue corresponded with increased synapsin-1 and decreased PSD-95 protein levels. These results demonstrate that temporary microglia depletion treatment after aversive exposure could serve as a powerful therapeutic countermeasure for prevention of radiation-induced memory deficits during space missions.

Recognition memory refers to the ability to judge a previously encountered item as familiar and depends on the integrity of the medial temporal lobe^27^. Perhaps the best known task to measure recognition memory in rodents is the NOR (also known as the “visual paired-comparison task” in studies with humans and monkeys) and relies on hippocampal function^28^. This behavioral task measures recognition memory without the use of aversive stimuli (shocks, water, noise) and is based on the natural tendency of rodents to explore novel situations and objects. It also requires little investigator interaction, thereby limiting potential interference in the output. Here we used the NOR task to investigate GCR-induced recognition memory deficits. We found that helium exposure does not impact recognition memory early (18 + days, Supplemental Fig. 1) after exposure. In line with other reports investigating microglia depletion for therapeutic purposes^21,29^, while microglia are depleted we do not detect any memory deficits. However, both 15 cGy or 50 cGy of whole body helium exposure cause deficits in recognition memory measured 90 + days post exposure, (Fig. 2A).

Current estimates suggest that a Mars voyage will last between 1.5–3 years, thus radiation exposure and associated radiation-induced deficits are an important problem facing astronauts over the entirety of their Mars journey^4^. We and others have previously demonstrated persistent radiation-induced deficits after exposures to multiple components of GCR such as protons^4^, iron^6,30,31^, silicon^8^, proton + iron exposure^4^, titanium or oxygen exposure^9–11^. A previous report investigated helium irradiation in rats and it found no deficits in NOR at low doses (0.1–10 cGy)^32^, however, the report was based on helium exposure confined to the head only. It is also important to note that radiation exposure on deep spaces missions will not be confined to individualized body regions. To this extent previous results from Rabin *et al*. comparing the effects of irradiating head only, body only and whole body with ^16^O ions demonstrated that whole-body exposures are the most deleterious to cognitive performance^33^. The results of this study and previous reports^9,12,32^ demonstrate that multiple particles found in GCR, including helium, can induce persistent deficits in recognition memory. Cognitive deficits are detectable over extended periods of time post exposure (90 + days) and suggest that charged particle exposure could pose significant problems for long duration missions’ success and quality of life of astronauts upon return to earth.

Temporary microglia depletion (via PLX) seven days after radiation exposure completely prevents the development of deficits in recognition memory. We only tested PLX beginning at seven days after radiation exposure due to experimental limitations (time and logistics associated with shipment between facilities). However, the fact that PLX was not given preventatively or immediately after exposure is significant for two main reasons. First, these results highlight the potential of temporary microglia depletion for treatment purposes. Having a therapeutic intervention with a broad treatment window is advantageous. However, we acknowledge there are additional experiments needed prior to administration in astronauts. Second, these findings address basic mechanisms of radiation-induced cognitive decline. It is clear that helium exposure can regulate microglia activity long-term (Figs 3,5 and 6) while not impacting microglia number at 90 + days post exposure (Fig. 4). We hypothesize that radiation-induced microglia alterations primarily occur acutely after injury (within the first three weeks) as we found that a temporary depletion of microglia with PLX from day 8–22 after helium exposure was sufficient to prevent long-term memory problems. While this may not be the only useful treatment window, it definitely represents an effective one. The repopulated microglia in irradiated animals display phenotypes different from the microglia in irradiated animals 90 + days later, (Figs 5 and 6**)**. Importantly, following completion of the PLX diet, microglia repopulate to numbers comparable to those of untreated animals, (Fig. 4**)**. Thus, a key difference between the 50 cGy and the 50 cGy + PLX group is the phenotype of the repopulated microglia suggesting that these cells are an important functional component of helium radiation-induced cognitive deficits.

PLX targets colony-stimulating factor 1 (CSF-1) which is crucial for the survival, differentiation, and proliferation of monocytes and microglia. The CSF-1/CSF-1R signaling pathway plays a pivotal role during early brain development. CSF-1-(null) mutant mice have reduced tissue macrophages and abnormal brain development^34,35^, and CSF-1R knockout mice are lethal before puberty^36^. However, depletion of microglia by CSF-1R inhibitors in adult mice causes no noticeable cognitive deficits (Fig. 2)^17,18,22,23,29^. Close to full depletion of microglia can be achieved by oral administration of CSF-1 inhibitor via food^22^, and after the inhibitor is withdrawn, microglia precursors in the CNS quickly proliferate, repopulate, and distribute to the entire brain^22,23^. We and others have recently demonstrated that partial or full depletion of microglia prevents chronic cognitive deficits induced by whole-brain cesium and X-ray irradiation^17,18^. Interestingly, in Alzheimer’s mouse models, depletion of microglia prevents neuronal loss, dendritic spine loss and cognitive deficits during the disease development^21,29^. Furthermore, in a diphtheria toxin-induced neuronal loss model, depletion of microglia was able to resolve inflammatory responses and promote brain recovery^37^. Taken together, these studies suggest that microglia depletion during or shortly after pathological-, physiological-, or chemical-induced brain damage can prevent the consequent neuronal deficits. However, the underlying mechanisms are still not clear. Although Elmore *et al*. showed that in the normal CNS, repopulated microglia have inflammatory expression profiles indistinguishable from those of normal non-depleted microglia^23^, this may not be the case during pathological states. In this study, we show for the first time that temporary microglia depletion can mitigate the adverse effects on recognition memory after exposure to space-like radiation. We demonstrate that the repopulated microglia elicit lower levels of inflammatory chemokines and cytokines after charged particle irradiation, (Fig. 3). These changes corresponded to the reduced complement receptor C5aR expression on microglia, (Fig. 5). This finding is in line with previous reports showing improved cognitive performance after C5aR antagonist treatment in different rodent models^38,39^.

The repopulated microglia have reduced lysosome membrane protein LAMP-1, indicating that they had lower phagocytic activity and, were therefore functionally distinct from their non-depleted brain counterparts. In our previous study we observed that microglia depletion prevented the dramatic reduction in spine density induced by cranial irradiation. Notably, microglia depletion alone did not cause changes in dendritic spine density in hippocampal granule neurons^18^. Given the established links between phagocytic microglia, complement activation and synapse depletion^24–26^ it is possible that by repopulating microglia and preventing the phagocytic phenotype we also block synapse and cognitive loss. In support of this possibility we observed changes in synapse stability following temporary microglia depletion. Specifically, we measured increases in the pre-synaptic protein synapsin-1 and decreases in the post-synaptic protein, PSD-95 in the 50 cGy + PLX group when compared with the charged particle exposure alone, (Fig. 7). Both synapsin-1 and PSD-95 are involved in synapse stability and function^40,41^ thus changes in these proteins is indicative of alterations in overall neuronal function consistent also with the behavioral changes observed here. Initial studies focusing on microglia and synapse interaction found that microglia primarily phagocytose presynaptic terminals^25^. This is in line with our finding that the less phagocytic repopulated microglia (in the 50 cGy + PLX group) could lead to more presynaptic protein expression. A previous report found that GCR exposure alone increases PSD-95 levels which corresponded with decreased dendritic spine density^9^. These alterations in the post-synaptic terminals correlated with radiation-induced behavioral deficits (NOR and object-in-place). The direct interaction between microglia phenotype and synapses after cosmic radiation exposure needs further investigation; nevertheless, this study provides an initial insight into potential mechanisms of transient microglia depletion and rescue of cognitive decline.

Understanding how radiation exposure impacts cognitive function is critical not only for astronauts on long missions but also for patients undergoing cancer therapy. Therapeutic whole brain irradiation usually consists of a total dose of 55–60 Gy delivered in 25–30 fractions. It causes a wide range of deleterious responses, including deficits in neural regeneration, damage to the blood-brain-barrier, activation of glial cells and infiltration of peripheral immune cells, that together may account for long-term cognitive impairment^42–46^. In the current study, we found significant reductions in the inflammatory chemokine CCL2 and the scavenger receptors CD206 and CD163 measured in the hippocampus of microglia depleted mice (50 cGy + PLX) compared to irradiated mice (50 cGy) 90 plus days post exposure. These data were also corroborated by the flow cytometry analysis demonstrating that the repopulated microglia have reduced lysosome protein LAMP-1. These results demonstrate that, after GCR exposure, microglia depleted brains have a less inflammatory and less stressful microenvironment than the microglia non-depleted brains. Together with our previous data in preclinical animal models of therapeutic irradiation, the results reported here further demonstrate the critical role that microglia play in the long-term development of radiation induced cognitive deficits^18,47^.

Microglia depleted brains after GRC show also a reduction in DUSP1, an upstream suppressor of the BDNF signaling suggesting that PLX-mediated microglia depletion may cause an indirect up-regulation of the microglial BDNF signal, which could be responsible for preserved cognitive outcomes^48,49^. Interestingly, we only found decreased DUSP1 expression in the 50 cGy + PLX group and not the 100 cGy + PLX group when compared to the GRC exposed animals without microglia depletion. We did not identify changes in CCL2 expression when comparing the 100 cGy and 100 cGy + PLX group. These results further imply that the behaviorally impaired group (50 cGy group) has a different inflammatory phenotype than animals in which no behavioral deficits are measured (50 cGy + PLX, 100 cGy and 100 cGy + PLX). We did however still see a modest decrease in CD163 and an increase in CD206 in the microglia-depleted brains mice when compared to irradiated only (100 cGy). These results suggest that changes in the expression of the scavenger receptor CD163 and the pattern recognition receptor CD206 in the repopulated microglia are not involved in loss of cognitive functions since the 100 cGy group did not show any cognitive deficits. Taken together, these results further strengthened the link between radiation induced microglia activation and its role on the development of cognitive functions.

In conclusion, our results provide potential mechanistic evidence for the role of microglia in the development of long-term cognitive deficits after cosmic radiation exposure. To our knowledge this is the first report to identify a therapeutic approach for treating GCR-induced deficits. Understanding how radiation exposure impacts cognitive function is critical not only for astronauts on long missions but also for patients undergoing cancer therapy.

## Methods

### Animals and whole body helium radiation exposure

All experiments were conducted in accordance with National Institutes of Health Guide for the Care and Use of Laboratory Animals and were approved by the Institutional Animal Care and Use Committee of University of California (San Francisco). Twenty-one week old C57B6/J wildtype (WT) mice were purchased from Jackson Laboratory (Bar Harbor, ME) and shipped to Brookhaven National Laboratory (BNL), Upton, New York where, after 1 week of acclimation, they were irradiated in an unmodulated beam of 250 MeV/n helium ions during two experimental campaigns at the NASA Space Radiation Laboratory as part of experiment # N301. These were NSRL 16B (mice irradiated 6/8/2016, at 1:30–3:30 PM) and NSRL 17A (mice irradiated 5/2/2017, at 1:40–1:56 AM). The mice were housed in groups of 4 on a normal 12:12 light cycle at the BNL animal care facility (provided food and water ad libitum), transported to the NSRL for irradiation several hours before exposure and returned to the animal care facility several hours after irradiation. In each case, mice were loaded into 7.3 × 4.0 × 4.0 cm polystyrene restraint boxes with air holes and mounted on the beam line either in a polyethylene foam adaptor (NSRL 16B) or as stacks of restraint boxes on a foam base (NRL 17A). After irradiation the animals were returned to their home cages and returned to the animal care facility for shipment to University of California San Francisco by World Courier™ after 2 or 5 days. For the NSRL 16B set, animals were oriented transverse to the 20 × 20 cm beam while during NSRL 17A the beam intensity was lower necessitating orientation of the animals parallel to the beam direction due to time restrictions. The beam energy was 249.3 MeV/n with a range in water of 36.6 cm and a predicted LET of 1.57 keV/µm. GERM transport code calculations indicate that at the target surface, fragments (Z = 0 or 1) accounted for 2% of the particle fluence while at the midline of transverse oriented animals fragments represented 5.1% of the fluence and for parallel oriented animals fragments represented 7.7% of the fluence at the midpoint [GERM reference]. For all depths the dose averaged LET was 1.57 while fluence- or event-based LET varied from 1.54 to 1.55 LET/µm. For NSRL 16B animals the exposures were: 15.007 ± 0.005 cGy (mean and standard deviation) with a dose rate of 16.37 cGy/min; 50.010 ± 0.004 cGy at 16.95 cGy/min and 100.007 ± 0.007 cGy at 18.07 cGy/min. For NSRL 17A there was one exposure each at 50.004 cGy and 100.002 cGy with an average dose rate of 19.98 cGy/min. Poisson distribution calculations indicate that the average number of particles traversing a 100 µm2 cellular target (≈ neuronal soma) are 60, 199 or 398 at 15, 50 and 100 cGy, respectively. In resting microglia, for which average soma area is ≈39 µm2 in C57Bl/6 mouse cortex^50^, the average fluence would be 23, 75 and 155 at 15, 50 and 100 cGy. This indicates that all relevant cells received multiple ion traversals and that dose was uniform throughout the cells and tissue.

### PLX treatment

Upon arrival at UCSF (seven days after radiation exposure), diets were switched to either PLX5622 or control diet (free base PLX5622 was provided by Plexxikon Inc, Berkeley, CA, and formulated in AIN-76A standard chow at 1200ppm by Research Diets Inc). Mice were group housed in environmentally controlled conditions with reverse light cycle (12:12 h light:dark cycle at 21 ± 1 °C) were provided diets and water ad libitum. Approximately, 1.2 mg of PLX5622 was ingested by a mouse of 25 grams of weight. Diets were switched to their regular diet after 14 days of treatment.

### Behavioral analysis

For all behavioral assays the experimenters were blinded both to the radiation exposure and diet treatment. One week prior to behavioral analysis, animals were handled for habituation to experimenters and room settings. Behaviors were performed in dark rooms during the animals wake cycle. All behaviors were recorded using an overhead camera connected to a video tracking and analysis system (Ethovision XT 12.0, Noldus Information Technology). When tracking was not optimal, videos were manually scored by an investigator blinded to radiation exposure and diet treatment. Animal behaviors were run on both irradiated cohorts (NSRL16B 12 mice/group; NSRL 17A 4/mice group- 0, 50, 100, 50 + PLX, 100 + PLX only). Total animal group numbers: 0 cGy = 15; 15 cGy = 12 15 cGy + PLX = 12; 50 cGy = 15; 50 cGy + PLX = 14; 100 gGy = 18; 100 cGy + PLX = 18. No behavioral differences between NSRL16B and NSRL17A were observed.

#### Novel object recognition

Memory function was measured both during PLX or control diets (days 18–21) and at late time points (90 + days), Fig. 1, by novel object recognition assay (NOR)^51^. This tool is frequently used to accurately reflect even mild deficits after radiation exposure^7,9,52–55^. The test environment consists of an open field arena (30 cm^2^) under red lighting. Mice were allowed to explore the arena for two 10-minute periods for two consecutive days (habituation phase). On day three (training phase), two identical objects (red Lego™ blocks) were secured to the floor in opposite corners of the arena using magnets and mice were allowed to explore the arena and objects for 5 minutes. 24 hours later on day four (testing phase), one of the objects was replaced with a novel object (orange Lego™ flower) of similar dimensions and texture. Mice were allowed to explore for 5 minutes. The objects and arena were cleaned with 70% ethanol between trials and animals. Trials were recorded and exploratory behavior was defined as time the animals spent directing its nose towards an object. Data is expressed as percent of time mice spent exploring each object. Mice that had less than 5 seconds of exploration time during either training or testing were excluded from analysis.

#### Anxiety

Anxiety was evaluated using the Elevated Plus Maze (EPM) at 90 days post helium exposure. The EPM consists of two exposed, open arms (35 cm) opposite each other and two enclosed arms (30.5 cm) also across from each other. The four arms are attached to a center platform (4.5 cm square) and the entire maze elevated 40 cm off the floor. Bright white lights are illuminated on both ends of the open arm^56^. Mice were placed individually onto the center of the maze and allowed to explore the maze for 5 minutes and their activity was recorded. The maze was cleaned with 70% ethanol between animals. Anxiety-like behavior was calculated by the percent time spent in the open arms + center.

#### Tissue collection

All mice were lethally overdosed using a mixture of ketamine (10 mg/ml) and xylaxine (1 mg/ml). Once animals were completely anesthetized, the chest cavity was opened and blood was obtained by cardiac puncture. Following cardiac puncture animals were perfused with 1 × phosphate buffer solution (PBS), pH 7.5 (Gibco, Carlsbad, CA, 70011–044).

For frozen tissue, following PBS, the whole brain was rapidly removed and the hippocampus and prefrontal cortex were dissected, snap frozen on dry ice and stored at −80 °C.

For flow cytometry analysis: After perfusion, brains from NSRL17A were quickly removed and cut into approximately 1 mm^3^ cubes by a razor blade and digested in 2 ml of cold Accutase in the the presence of DNase I (Sigma Aldrich, DN25-100MG) on ice with frequent pipetting for 20 minutes. Cells suspension was then passed through a 70um cell strainer to remove large debris. Cells were collected by centrifugation and then resuspended in 25% Percoll™ solution (Sigma, P4937-100ML) diluted in RPMI medium. After centrifuging at 800 × g for 20 minutes at 4 °C, myelin debris and excess solutions were discarded. Cell pellet was then washed with fresh RPMI medium and resuspended in 100ul FACS buffer (1 × DPBS with 0.5% BSA fraction V, Gibco 15260037). Cells were blocked with one volume of blocking solution (5% normal mouse serum, 5% normal rat serum, 5% normal rabbit serum (Jackson ImmunoResearch, 015-000-120, 012-000-120, 011-000-120), 2% FBS (Gibco, 10082139), and 1% BSA fraction V × 1 DPBS) for 30 minutes, stained for 30 minutes with fluorophore-conjugated antibodies on ice (CD11b-AF700, 1:100, CD45-FITC 1:50, BD Pharmingen 557690 and 553080; C5aR-PE, 1:100, Miltenyi Biotech 130-106-174). For intracellular staining, cells pre-stained with extracellular markers were fixed in 500ul Fixation/Permeabilization solution (BD Biosciences 554714) on ice 15 minutes, washed twice with 1 × perm/wash buffer (BD Biosciences 554714) and stained with intracellular markers on ice for 30 minutes (CD107a-PE, 1:200, Miltenyi Biotec 130-102-219; CD206-PE, 1:50, Biolegend 141706). Stained cells were washed and resuspended in FACS buffer before analyses. Data were collected on an Aria III sorter (BD) and analyzed with Flowjo™ software (v10, Tree Star Inc.). At least 3,000 microglia events (CD11b^+^CD45^low^) were collected from each brain for fluorescence intensity comparisons.

#### Gene array analysis and qPCR confirmation

Dissected ipsilateral hippocampi from NSRL 16B cohorts were used for all gene expression analyses. RNA isolation and cDNA conversion were completed as previously described^57,58^. RNA concentration and quality were determined using a NanoDrop (Thermo Scientific). Three hundred nanograms of RNA was reverse transcribed using High-Capacity cDNA Reverse Transcription Kit (Applied Biosystems). For inflammatory profiling arrays^59^, (Qiagen, #330131) equal volumes of cDNA for each sample were pooled (n = 11 animals/group/pool) and run on a single plate per condition; cycling conditions were followed as suggested by manufacturer. Inflammatory genes analyzed summarized in Supplemental Table 2. Select analytes from the profiling arrays were validated using individual samples (n = 11/group) carried out in duplicate using SYBR Green Master Mix (Applied Biosystems) following manufacturer’s suggested protocol. The relative expression of target genes was determined by the 2−ΔΔCt method and normalized against beta-actin gene expression using a Statagene Mx3005P Real-Time PCR system. 50 cGy and 50 cGy + PLX groups were standardized to 0 cGy group. 100 cGy and 100 cGy + PLX were standardized to 100 cGy group. The primers used were:

CCL2: 5′: GCTGACCCCAAGAAGGAATG-3′

CD206: 5′ CCTCTGGTGAACGGAATGAT-3′

DUPS1: 5′-CAACCACAAGGCAGACATCAGC-3′

CD163: 5′-GCTAGACGAAGTCATCTGCACTGGG-3′.

Genes tested for differential expression in which significance was not reached: CCL7, GATA3, GMCSF, IL-6 and CD36.

#### Western blot analysis

Snap frozen prefrontal cortex tissues NSRL 16B cohorts were hand homogenized in Pierce RIPA buffer (Thermo Scientific, #89900) with cOmplete ULTRA tablets (Roche, #05892791001) and PhoSTOP (Roche, #04906837001) protease inhibitors for whole cell lysis. Debris was removed via centrifugation and the protein concentration of the remaining homogenate was measured using the Piece BCA Protein Assay (Thermo Scientific, #23225) following the manufacturer’s instructions. 20 ug of protein in Laemelli buffer (BioRad, #1610747) with beta mercaptoethanol (Sigma, M-3148) was boiled at 100 °C, loaded into a precast 4–15% Tris Glycine gel (BioRad, #5671084), and run using Tris/Glycine/SDS buffer (BioRad, 1610772) at 150 V. Protein was transferred onto a nitrocellulose membrane (BioRad, 1620168) using Tris/Glycine buffer (BioRad, 1610771) at 100 V. Membranes were blocked using Odyssey Blocking Buffer (PBS) (LI-COR, 92740000) and incubated for a minimum of 14 hours with either synapsin 1 (1:2000 in PBS, Millipore, #AB1543) or PSD95 (1:1000 in PBS, Abcam, #ab13552). Blots were washed using TBS and incubated for 60 minutes in their host-specific secondary antibody (LI-COR, #92632210-Mouse, #92632211-Rabbit). Blots were imaged using Odyssey (LI-COR) imaging system and quantified using the Image Studio software. All blots were normalized to GAPDH (Sigma, #G8795).

#### Data analysis

Results were analyzed using Prism software (v7.05, GraphPad™; La Jolla, CA) and expressed as mean ± standard error of the mean (SEM). Statistical analyses were performed as listed below with p values of <0.05 considered as significant.

Novel Object Recognition: Two-way repeated measure ANOVA, Sidak post-hoc analysis.

Exploration time: One- way ANOVA; no post hoc analysis because significance was not reach.

Elevated Plus Maze: One- way ANOVA; no post hoc analysis because significance was not reach.

qPCR: Unpaired Students t-test.

Flow Cytometry: Unpaired Student t-test.

Western Blot analysis: Unpaired Students t-test.

## Electronic supplementary material

Supplemental Information
