# Author Correction: Temporary microglia-depletion after cosmic radiation modifies phagocytic activity and prevents cognitive deficits

**DOI:** 10.1038/s41598-018-28390-1

**Published:** 2018-07-03

**Authors:** Karen Krukowski, Xi Feng, Maria Serena Paladini, Austin Chou, Kristen Sacramento, Katherine Grue, Lara-Kirstie Riparip, Tamako Jones, Mary Campbell-Beachler, Gregory Nelson, Susanna Rosi

**Affiliations:** 1 Department of Physical Therapy and Rehabilitation Science, University of California, San Francisco, CA USA; 2 Brain and Spinal Injury Center, University of California, San Francisco, CA USA; 3 Department of Basic Sciences, Division of Biomedical Engineering Sciences, Loma Linda University, Loma Linda, CA USA; 4 Department of Neurological Surgery, University of California, San Francisco, CA USA; 5 Weill Institute for Neuroscience, University of California San Francisco, San Francisco, CA USA; 6 Kavli Institute of Fundamental Neuroscience, University of California San Francisco, San Francisco, CA USA

Correction to: *Scientific Reports* 10.1038/s41598-018-26039-7, published online 18 May 2018

This Article contains typographical errors in the Acknowledgements section.

“This work was supported by NASA grant NNX13AD60G.”

should read:

“This work was supported by NASA grant NNX14AC94G.”

