# Supplementary material for: Temporary microglia-depletion after cosmic radiation modifies phagocytic activity and prevents cognitive deficits
Source: Sci Rep. 2018 May 18;8:7857. doi: 10.1038/s41598-018-26039-7 (PMC5959907; doi:10.1038/s41598-018-26039-7)
Supplement: Supplementary file 1 — Supplemental Information [file 41598_2018_26039_MOESM1_ESM.pdf]

Title: Temporary microglia-depletion after cosmic radiation modifies phagocytic activity and prevents cognitive deficits

Authors: Karen Krukowski*[1,2], Xi Feng*[1,2], Maria Serena Paladini[1,2], Austin Chou[1,2], Kristen Sacramento[1,2], Katherine Grue[1,2], Lara-Kirstie Riparip[1,2], Tamako Jones[3], Mary Campbell-Beachler[3], Gregory Nelson[3] and Susanna Rosi[# 1,2,4,5,6]

[1] Department of Physical Therapy and Rehabilitation Science, University of California, San Francisco, CA, USA.

[2] Brain and Spinal Injury Center, University of California, San Francisco, CA, USA.

[3] Department of Basic Sciences, Division of Biomedical Engineering Sciences, Loma Linda University, Loma Linda, CA,USA.

[4] Department of Neurological Surgery, University of California, San Francisco, CA, USA.

[5]Weill Institute for Neuroscience, University of California San Francisco, CA, USA.

[6] Kavli Institute of Fundamental Neuroscience, University of California San Francisco, CA, USA.

Corresponding Author:

Susanna Rosi, Ph.D.
1001 Potrero Ave,
Zuckerberg San Francisco General Hospital
Building#1 room 101
San Francisco, CA 94110
Tel.: +415-206-3708
Email: susanna.rosi@ucsf.edu

*authors contributed equally

Running title:
Microglia and cognition after space radiation

Supplemental Figure 1. **Investigation of acute radiation-induced memory impairments**. Animals were exposed to helium radiation (0, 15, and 50, 100 cGy). Beginning at 18 days post radiation, animals were tested for memory deficits. Animals were on either PLX or control diets during testing. Memory deficits were measured by novel object recognition. Animals were exposed to two identical object, 24 hrs later the animals are exposed to one familiar object and one novel object. Memory deficits are calculated by a deficit in distinguishing the new object. Nv= novel. Fm= Familiar. Two-way repeated measured ANOVA found a significant discrimination effect ($p<0.0001$). Sidak post hoc analysis revealed differences in discrimination effects. *** p,0.001. Individual animal scores represented in dots, bars depict group mean and SEM.

Supplemental Figure 2. **Western blot analysis of neuronal stability markers.** Western blot analysis of isolated prefrontal cortex tissues revealed changes in neuronal stability markers: synapsin 1 (**A**) and PSD95 (**B**).

Supplemental Table 1. Inflammatory gene expression levels in 100 cGy and 100 cGy + PLX groups. qPCR analysis to measure expression differences between the 100 cGy and 100 cGy + PLX groups. Standardized to 100 cGy group. Four genes were measured (CCL2, CD206, DUSP1 and CD163). Unpaired Student t-test revealed significant differences between groups. * $p<0.05$, ** $p<0.01$. Group mean and SEM listed. N=11-12 each group.

Supplemental Table 2. Array genes list. The individual genes analyzed in the array in Figure 3 are listed in the order in which the genes appear on the heat map in Figure 3A.

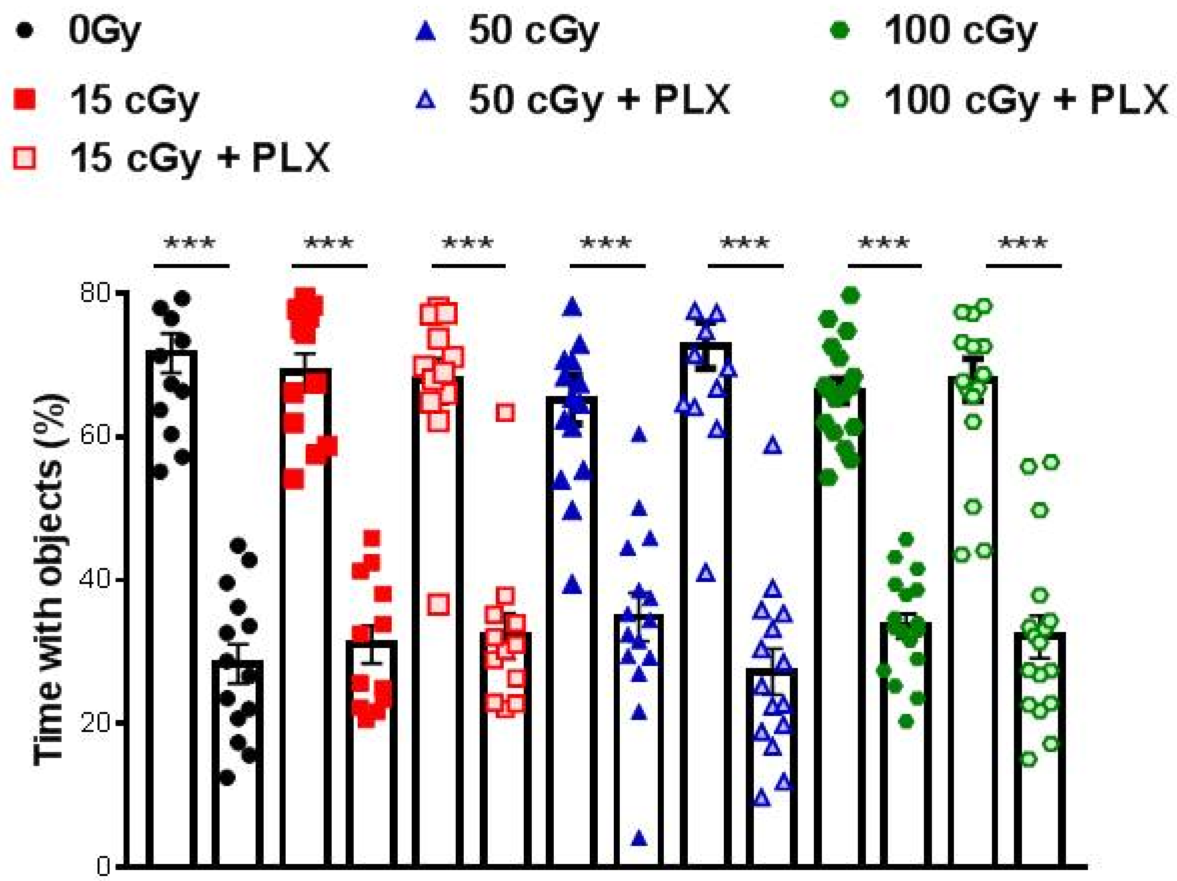

0Gy
50 cGy
100 cGy
15 cGy
50 cGy + PLX
100 cGy + PLX
15 cGy + PLX
***
***
***
***
***
***
***
80
60
40
20
0
Time with objects (%)

A

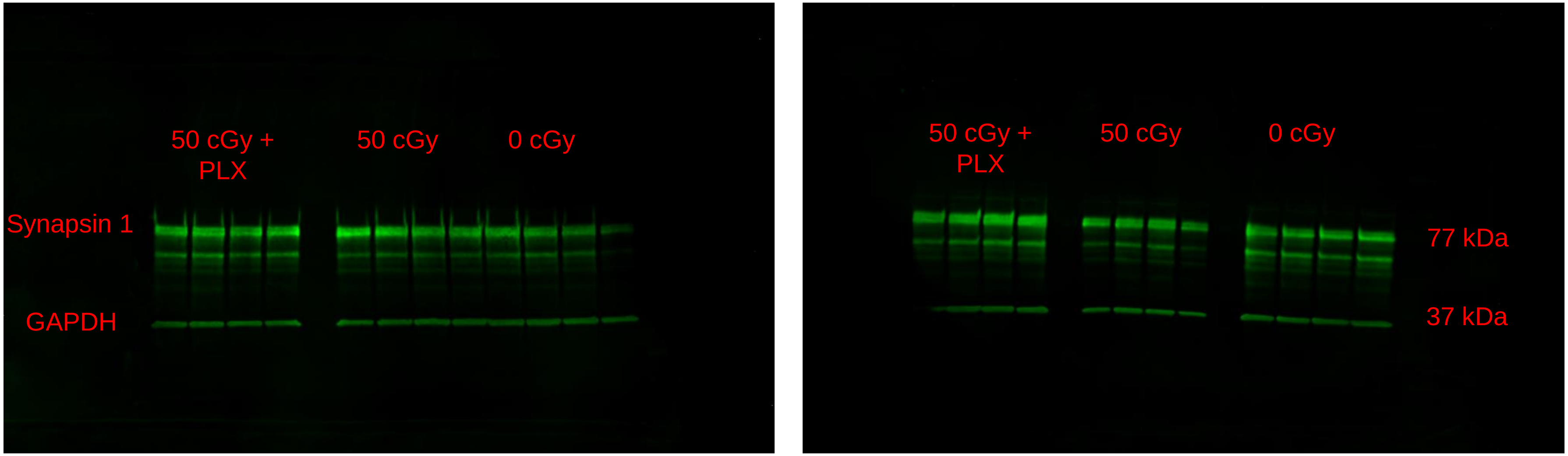
50 cGy +
PLX
50 cGy
0 cGy
Synapsin 1
GAPDH
50 cGy +
PLX
50 cGy
0 cGy
77 kDa
37 kDa

B

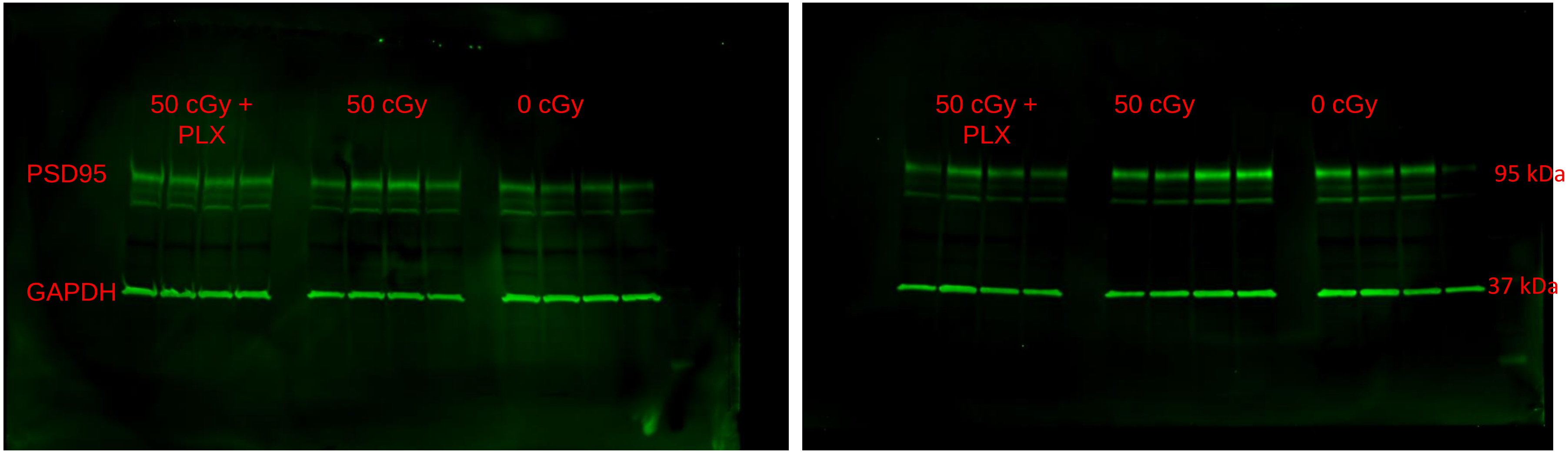
50 cGy +
PLX
50 cGy
0 cGy
PSD95
GAPDH
50 cGy +
PLX
50 cGy
0 cGy
95 kDa
37 kDa

Treatment Group	CCL2	CD206	DUSP1	CD163
**100 cGy**	1.19 ± 0.1	1.0 ± 0.1	1.01 ± 0.0	1.08 ± 0.1
**100 cGy + PLX**	0.92 ± 0.1	1.68 ± 0.1**	0.94 ± 0.3	0.57 ± 0.1*

	Gene	
0	Cxcl9	
1	Cd40lg	
2	TLR1	
3	Ccl17	
4	Cxcl10	
5	Ccl1	
6	Ccl12	
7	Tlr7	
8	Cxcl2	
9	Cxcl15	
10	Il10	
11	DC-Sign	Upregulated
12	gmcsf	
13	Ccl24	
14	Cxcl3	
15	PTX3	
16	Stat3	
17	Il6	
18	MARCO	
19	Cxcl11	
20	Cxcl5	
21	Ccl20	
22	Cxcl13	
23	CD206	
24	CD36	
25	IRF4	
26	MMP2	
27	IL25	
28	LIF	
29	Tnfa	
30	Ccl5	
31	Ccl8	
32	SLAM	
33	Cd86	
34	Ccl22	
35	VEGF	
36	Stat6	
37	Cd40	
38	Ifng	
39	Gapdh	
40	NOS2	
41	mcsf	
42	PPARg	

43	TIMP3	
44	Actb	
45	Arg1	
46	IL4Ralpha	
47	Cxcl12	
48	Mif	
49	Cd14	
50	TLR2	
51	IRF5	
52	Gata6	
53	Stat1	
54	Cxcl14	
55	Jak2	
56	Ccl3	
57	Cxcl16	
58	Tlr3	
59	TLR4	
60	YM1	
61	IRF8	
62	jmjd3	
63	Fpr1	
64	Il15	
65	Ccl6	
66	Cxcl1	
67	Il1b	
68	Ccl4	
69	IL33	
70	MMP9	
71	TLR8	
72	Tlr9	
73	Cx3cl1	
74	Tgfb1	
75	BMP2	
76	Stat5	
77	Gata3	Downregulated
78	Ccl7	
79	CD163	
80	Ccl2	
81	Cd80	
82	Il13	
83	Ccl19	
84	TIMP1	
85	Ccl9	
86	Fizz1	

87	Il16	
88	Ccl11	
89	Il4	
90	DUSP1	
91	Ccl25	
92	TGM2	
